# Living or deceased-donor kidney transplant: the role of psycho-socioeconomic factors and outcomes associated with each type of transplant

**DOI:** 10.1186/s12939-020-01200-9

**Published:** 2020-06-01

**Authors:** Abbas Basiri, Maryam Taheri, Alireza Khoshdel, Shabnam Golshan, Hamed Mohseni-rad, Nasrin Borumandnia, Nasser Simforoosh, Mohsen Nafar, Majid Aliasgari, Mohammad Hossein Nourbala, Gholamreza Pourmand, Soudabeh Farhangi, Nastaran Khalili

**Affiliations:** 1 Urology and Nephrology Research Center (UNRC), Shahid Labbafinejad Medical Center, Shahid Beheshti University of Medical Sciences, Tehran, Iran; 2 Urology and Nephrology Research Center (UNRC), Shahid Beheshti University of Medical Sciences, Tehran, Iran; 3 Modern Epidemiology Research Center, Aja University of Medical Sciences, Tehran, Iran; 4 Department of Urology, Ardabil University of Medical Sciences, Ardabil, Iran; 5 Chronic Kidney Disease Research Center, Urology and Nephrology Research Center, Shahid Labbafinejad Medical Center, Shahid Beheshti University of Medical Sciences, Tehran, Iran; 6 Baqiyatallah University of Medical Sciences, Tehran, Iran; 7 Urology Research Center, Ibin Sina Medical Center, Tehran University of Medical Sciences, Tehran, Iran; 8 Shahid Beheshti University of Medical Sciences, Tehran, Iran

**Keywords:** Deceased donor, Living donor, Kidney transplant, Psychologic status, Socioeconomic

## Abstract

**Background:**

Kidney transplant improves patients’ survival and quality of life. Worldwide, concern about the equality of access to the renal transplant wait-list is increasing. In Iran, patients have the choice to be placed on either the living or deceased-donor transplant wait-list.

**Methods:**

This was a prospective study performed on 416 kidney transplant recipients (*n* = 217 (52.2%) from living donors and *n* = 199 (47.8%) from deceased donors). Subjects were recruited from four referral kidney transplant centers across Tehran, Iran, during 2016–2017. The primary outcome was to identify the psycho-socioeconomic factors influencing the selection of type of donor (living versus deceased). Secondary objective was to compare the outcomes associated with each type of transplant. The impact of psycho-socioeconomic variables on selecting type of donor was evaluated by using multiple logistic regression and the effect of surgical and non-surgical variables on the early post-transplant creatinine trend was assessed by univariate repeated measure ANOVA.

**Results:**

Based on standardized coefficients, the main predictors for selecting living donor were academic educational level (adjusted OR = 3.25, 95% CI: 1.176–9.005, *p* = 0.023), psychological status based on general health questionnaire (GHQ) (adjusted OR = 2.46, 95% CI: 1.105–5.489, *p* = 0.028), and lower monthly income (adjusted OR = 2.20, 95% CI: 1.242–3.916, *p* = 0.007). The waiting time was substantially shorter in patients who received kidneys from living donors (*p* < 0.001). The early post-transplant creatinine trend was more desirable in recipients of living donors (β = 0.80, 95% CI: 0.16–1.44, *p*-value = 0.014), patients with an ICU stay of fewer than five days (β = − 0.583, 95% CI: − 0.643- -0.522, p-value = < 0.001), and those with less dialysis duration time (β = 0.016, 95% CI: 0.004–0.028, p-value = 0.012). Post-operative surgical outcomes were not different across the two groups of recipients (*p* = 0.08), however, medical complications occurred considerably less in the living-donor group (*p* = 0.04).

**Conclusion:**

Kidney transplant from living donors was associated with shorter transplant wait-list period and better early outcome, however, inequality of access to living donors was observed. Patients with higher socioeconomic status and higher level of education and those suffering from anxiety and sleep disorders were significantly more likely to select living donors.

## Introduction

Currently, there is a global concern regarding inequality of access to the renal transplant waiting list [1]. Socioeconomic disparities influence the access to the kidney transplant wait-list in different parts of the world [1, 2]. In the United Kingdom and the USA, access to the transplant waiting list is limited for deprived patients, while in France, patients with a low socioeconomic status have an equal access [3–5]. Due to national insurance policies in Iran, complete cost coverage is not feasible for every patient, thus patients’ socioeconomic status plays an important role in donor type selection.

In Iran, patients in need of kidney transplants are placed on two separate wait-lists, based on the type of donor they select, living versus deceased donors. Thus far, studies have provided evidence that the outcome of living-donor transplant is superior to receiving kidneys from deceased donors [6–9]. This has led to the increasing attention and desire for recipients to opt for living unrelated donor transplants.

Several factors contribute to this preference including psychological and socioeconomic factors. Among psychological variables, anxiety and depression pose unique challenges in the course and prognosis of a disease, as well as its management [10–14]. The relatively prevalent coexistence of anxiety and depressive disorders with chronic medical conditions has diverse clinical consequences on treatment-seeking patterns and management strategies [15, 16]. Considering the fact that kidney transplant candidates have to wait for a considerably long time before receiving a graft, this anticipation makes them prone to psychiatric issues such as depression and anxiety [17–19].

Although Iran was the first country to establish a living unrelated donor program in 1988 and living kidney donation has been performed frequently since then, a multicenter analysis comparing the psycho-socioeconomic status of recipients of living and deceased donors is not yet available [20–22]. Moreover, although previous studies have shown the necessity of psycho-social screening in patients awaiting transplant for selecting the best potential recipients, there is lack of data regarding the role of psycho-socioeconomic factors on selecting the best type of donor [23]. Thus, the primary objective of this study was to investigate psycho-socioeconomic factors influencing patients’ decision of choosing type of donor in Iran. We also assessed the early outcomes associated with each type of transplant.

## Objectives

The primary outcome of this study was to identify the psychological and socioeconomic factors which influence the selection of type of donor (living versus deceased). The secondary objectives were to compare the early medical and surgical outcomes associated with each type of transplant, identify predictive factors of early post-transplant creatinine trend (as a marker of kidney function) and also estimate the mean waiting time for each type of kidney transplant while on the transplant wait-list.

## Methods

### Design and setting

This was a prospective cohort study conducted on patients undergoing kidney transplant regardless of etiology. Patients were recruited from one of the four transplant centers across Tehran including; Labbafinejad hospital, Baqiatallah hospital, Sina hospaital and Modarres hospital, during 2016–2017. Selection of hospitals was based on the following criteria:

- The center had to be a referral center located in Tehran, Iran.
- The center had to be a university hospital affiliated to one of the medical universities of Tehran, Iran.
- The staff of the center had to be willing to participate in this study and cooperate in sample collection.

### Study participants

Eligible subjects included patients aged ≥18 years old receiving a kidney transplant during 2016–2017, in one of the four centers mentioned above. A total of 416 recipients including 217 patients (52.2%) receiving grafts from living donors and 199 (47.8%) receiving deceased-donor transplants were included. The sampling approach was based on a three-month pilot study performed before the initiation of the actual study with the aim to estimate the number of transplants performed in each center and determine the dominant type of transplantation (deceased vs living) in that center. Then, based on probability proportional to size (PPS) sampling, the number of subjects required from each center was calculated. In the next step, sample recruitment started with both types of transplants. When the number of samples of the dominant type of transplant (in each specific center) reached approximately half of the required total sample size, no more samples of this type of transplant were included and recruitment would continue with the less frequent type of donor until the total sample size was reached. This ensured that within a particular timeframe, a proportionate number of living and deceased-donor transplant recipients would be included without intentionally or non-intentionally excluding any cases. Also, the adjustment of the proportion of living and deceased kidney transplant recipients allowed for a more reliable comparison.

### Data collection

Medical files and questionnaires were used to collect demographic, psycho-socioeconomic, and clinical data of patients before kidney transplant. Data regarding post-transplant clinical outcome of patients (medical and surgical) was collected by an independent investigator on day 1, 3, 5, 7, and 11 after transplant and eventually on the day of discharge based on history taking, physical examination, lab data, imaging and medical records. The indicators of socioeconomic position included employment status, health insurance, financial support, pre-dialysis income and education level of recipients one year before dialysis start. Time to kidney transplant was defined as time from placement on the waiting list to the date of the transplant. Income was calculated as individual (after-tax) disposable income including from work and benefits and was categorized according to the consensus of the Iranian Ministry of Cooperative, Labor, and Social Welfare. Financial support was considered as any financial assistance from family, relatives, or non-governmental organizations.

The translated General Health Questionnaire-28 (GHQ-28) was used for assessing mental symptoms and psychosocial well-being of the recipients [24]. According to Social Support Questionnaire (SSQ), three different source of support including family, friends, and significant others were evaluated [25]. Also, the translated 36-Item Short Form Health Survey questionnaire (SF-36) was applied to evaluate health-related quality of life [26]. Validity and reliability of the Persian version of these questionnaires has been approved in the Iranian population [27, 28].

### Statistical analysis

Continuous variables were presented as mean (standard deviation) and categorical variables were reported as frequencies (percentage). The independent t-test or Mann-Whitney were used to examine the association between quantitative variables, and Chi-Square or Fisher’s Exact test were used for assessing categorical variables. The impact of psycho-socioeconomic variables on selecting the type of donor were evaluated using the multiple logistic regression with backward approach. Results are reported as odds ratio (OR) with 95% confidence interval (CI) and *p*-values. Also, the importance of variables was determined by computing the standardized coefficient. Univariate repeated measure ANOVA assessed the effect of surgical and non-surgical variables on the early post-transplant creatinine trend. Subsequently, variables with a p-value less than 0.05 entered the multivariable generalized estimating equation (GEE) model. Finally, to identify predictive factors on the difference between serum creatinine before transplant and on the day of discharge, multivariate linear regression analysis was performed. All statistical analysis was performed using the SPSS software version 14.0 (IBM, Chicago, Illinois, USA) and also the ‘reghelper’ package in R software. *P*-value less than 0.05 was considered statistically significant.

### Ethical approval

Written informed consent was obtained from participants before being enrolled in the study. The Ethics Committee of the Urology and Nephrology Research Center of Shahid Beheshti University of Medical Sciences (ethics code: UNRC.SBMU.931223.1) approved this study.

## Results

Analysis was conducted on 416 recipients. Among the total participants, 68% (*n* = 248) were male. There was no statistically significant difference between the two groups of recipients in terms of gender (*p* = 0.34). The mean age of the patients transplanted with living and deceased donors was 42.56 (SD = 15.87) and 40.45 (SD = 16.0) years old, respectively (*p* = 0.13). The mean duration of dialysis was 14.7 (SD = 17.1) months in recipients of living and 17.9 (SD = 22.6) months in recipients of deceased donors (*p* = 0.11). Also, the mean wait-list period for kidney transplant was significantly shorter in recipients of living compared to deceased kidney donors (7.05 vs. 11.27 months, *p* < 0.001).

### Socioeconomic and psychological characteristics of the recipients

As shown in Table 1**,** recipients’ ethnicity, academic level of education, employment status, and monthly income were found to significantly influence the choice of selecting living or deceased donors (*p* = 0.008, 0.002, 0.002, < 0.001, respectively). Regarding the four main areas covered by the GHQ-28 questionnaire [24], anxiety and insomnia were reported to be higher among recipients of living donors (*p* = 0.044), while more patients receiving transplants from deceased donors suffered from depression symptoms (*p* = 0.045). Among the two distinct concepts of physical and mental health measured by the SF-36 questionnaire [29], there was no significant difference regarding the physical component summary (PCS) and the mental component summary (MCS) between living or deceased donor recipients (*p* = 0.435 and *p* = 0.788, respectively). Also, there were no difference between social support of the family, friends and significant others in the two groups of recipients based on the SSQ questionnaire. Table 1 shows results in detail.

**Table 1:** Demographic, psycho-socioeconomic and clinical characteristics of recipients based on living or deceased donor

	Living (***n*** = 217) N (%)	Deceased (***n*** = 199) N (%)	***p*** value
Age (years) ^§^			
**mean (SD)**	42.56 (15.87)	40.45 (16.00)	.13
**Gender**^†^			
Male	153 (70.5)	131 (65.8)	.34
Female	64 (29.5)	68 (34.2)	
**Ethnicity**^†^			.008
Fars	121 (55.7)	128 (64.32)	
Turk	43 (19.81)	40 (20.1)	
Kurd	21 (9.67)	5 (2.51)	
Arab	12 (5.52)	7 (3.51)	
Lur	6 (2.76)	11 (5.52)	
Other	14 (6.54)	8 (7.54)	
**Education**^‡^			.002
Illiterate	28 (13.3)	36 (18.7)	
Elementary or high school	60 (28.6)	57 (29.5)	
Diploma	71 (33.8)	80 (41.5)	
Academic degree	51 (24.3)	20 (10.4)	
Not declared	7 (3.22)	6 (3.01)	
**Employment**‡			.002
Full time	58 (29.7)	28 (15.6)	
Part time	16 (8.2)	19 (10.6)	
Self employed	22 (11.3)	10 (5.6)	
Unemployed	66 (33.8)	89 (49.7)	
Retired	30 (15.4)	27 (15.1)	
Disabled person	3 (1.5)	6 (3.4)	
Not declared	22 (10.1)	20 (10.0)	
**Monthly income**^**a** ‡^			<.001
Low income	81 (43.1)	122 (69.3)	
Lower-middle income	82 (43.6)	43 (24.4)	
Upper-middle income	25 (13.3)	11 (6.3)	
Not declared	29 (13.3)	23 (11.5)	
**Health Insurance Coverage**^†^			.185
Yes	213 (98.2)	191 (96.0)	
No	4 (1.8)	8 (4.0)	
**Financial support**^**b**†^			.486
Yes	83 (45.4)	81 (49.7)	
No	100 (54.6)	84 (50.9)	
**GHQ-28 questionnaire**			
Somatic symptoms	164 (42.3)	137 (35.3)	.162
Anxiety and sleep disorder	168 (43.3)	136 (35.1)	.044
Social dysfunction	79 (20.4)	81 (20.9)	.290
Depression symptoms	145 (37.5)	147 (37.9)	.045
**SF-36 questionnaire**			
mean (SD)			
Physical component summary	45.23 (16.24)	46.49 (15.36)	.435
Mental component summary	50.95 (25.97)	51.74 (31.16)	.788
**Social Support Questionnaire**^†^			
Family: Low or Moderate	23 (12.5)	27 (13.1)	.858
High	161 (87.5)	179 (86.9)	
Friends: Low or Moderate	73 (35.4)	60 (32.6)	.556
High	133 (64.4)	124 (67.4)	
Significant other: Low or Moderate	19 (10.4)	25 (12.4)	.552
High	163 (89.6)	177 (87.6)	
**ESRD causes**^†^			.22
Hypertension	77 (37.2)	54 (28.4)	
Diabetes Mellitus	26 (12.6)	34 (17.9)	
Glomerulonephritis	33 (15.9)	24 (12.7)	
Urologic diseases ^b^	23 (11.1)	30 (15.8)	
ADPKD	13 (6.3)	20 (10.5)	
Others ^c^/Unspecific	45 (21.0)	37(19.4)	

ESRD: end stage renal disease, ADPKD: autosomal dominant polycystic kidney disease

^a^ According to the consensus of the Iranian Ministry of Cooperative, labor, and social welfare, the minimum wage in 2016–2017 was approximately 10.000.000 Rials per month. Therefore, monthly income of recipients was divided into these tertiles: low income (monthly income: < 10.000.000 Rials), lower-middle income (between 10.000.000–20.000.000 Rials) and upper-middle income (> 20.000.000 Rials)

^b^ Financial support, any financial assistance from family, relatives, or non-governmental organizations

† Chi Square Test

‡ Mann Whitney

§ Independent sample T test

The results of unadjusted analyses to determine predictive factors of donor type selection are presented in supplementary Table 1. Also, Table 2 presents the results of adjusted analysis in detail. According to adjusted analysis, patients with academic education were almost 3.25 times more likely to select living-donors compared with the illiterate (OR = 3.41, 95% CI: 1.17–9.00). There was an almost 2.2 times higher probability for patients belonging to the lower-middle income group to select living-donor transplant compared with the low-income group (OR = 2.2, 95% CI: 1.24–3.91). Moreover, those with anxiety and sleep disorder were almost 2.46 times more likely to receive living-donor transplant compared with patients without anxiety and sleep disorder (OR = 2.46, 95% CI: 1.10–5.48). However, the GHQ4 was not significant in multiple logistic analysis, in unadjusted analysis those with depression symptoms were almost 40% less likely to receive living-donor transplant compared to those without depression symptoms. The other unadjusted results was similar and aligned with adjusted ones.

**Table 2:** Multiple logistic regression to determine predictive factors for selecting type of donor

	Adjusted OR	95% CI for OR		*p*-value
		Lower	Upper	
**Age**	1.012	.992	1.032	.231
**Gender (reference: male)**	.840	.440	1.604	.598
**Education (reference: illiterate)**				
Elementary to high school	.936	.427	2.054	.870
Diploma	1.121	.515	2.441	.774
Academic	3.254	1.176	9.005	**.023**
**Monthly income (reference: low income group)**				
Lower-middle income	2.205	1.242	3.916	**.007**
Upper-middle income	2.847	.937	8.651	.065
**Ethnicity (reference: other)**	.617	.357	1.066	.084
**Financial support (reference: yes)**	.655	.343	1.249	.199
**GHQ2 (reference: without anxiety and sleep disorder)**	2.462	1.105	5.489	**.028**
**GHQ4 (reference: without depression symptoms)**	.702	.335	1.471	.349
**SF-36: Physical component summary**	1.004	.979	1.029	.780
**SF-36: Mental component summary**	1.005	.989	1.022	.519
**SSQ family (reference: low + med)**	.684	.190	2.463	.562
**SSQ friend (reference: low + med)**	.950	.469	1.925	.888
**SSQ entourage (reference: low + med)**	.996	.267	3.715	.995
**ESRD causes (reference: DM + HTN)**	.905	.523	1.566	.722

OR, odds ratio. CI, confidence interval. DM, diabetes mellitus. HTN, hypertension. GHQ, General Health questionnaire. SF-36, Short Form Health Survey-36 SSQ, Social support questionnaire. ESRD, End stage renal disease

### Clinical outcomes of the kidney transplant

The most common reasons for receiving kidney transplant among the total population were hypertension and diabetes mellitus (31.5 and 14.4% of all cases, respectively) (Table 1**)**. Post-operative clinical status of all recipients including medical and surgical outcomes and laboratory tests is presented in Table 3. Post-operative surgical outcomes were not different across the two groups of recipients (*p* = 0.08), however, medical complications occurred considerably less in the living-donor group (*p* = 0.04).

**Table 3:** Clinical outcome of recipients based on type of donor

	Living (***n*** = 217)	Deceased (***n*** = 199)	*p*-value‡
	n (%)	n (%)	
**Medical events**			.04
Acute Rejection	13(6)	17 (8.5)	.60
ATN	36 (16.6)	54 (27.13)	.03
DGF	43 (19.8)	68 (34.17)	.004
Arrhythmia	7 (3.22)	7 (3.5)	.97
Myocardial infarction	0 (0)	7 (3.5)	.02
Pulmonary emboli	3 (1.38)	3 (1.5)	.99
Infection	9 (4.15)	6 (3.01)	.82
**Surgical events**			.08
Hemorrhage	10 (4.6)	8 (4.02)	
Vascular thrombosis	1 (0.46)	5 (2.51)	
Ureteral complication	3 (1.38)	3 (1.5)	
Surgical site infection	2 (0.92)	3 (1.5)	
**Lab Data** mean (SD)	Before Transplant→ After Transplant	Before Transplant→ After Transplant	p-value†
Hb	11.09 (2.28) → 10.05 (1.72)	11.54 (2.03) → 9.5 (1.49)	.04
WBC	10.40 (14.7) → 13.92 (13.20)	8.92 (7.26) → 10.39 (6.59)	.22
PLT	184.98 (61.8) → 189.61 (64.8)	196.95 (99.5) → 186.36 (66.0)	.15
AST	17.47 (9.11) → 26.60 (16.76)	20.04 (13.62) → 89.26 (7.65)	.03
ALT	19.22 (10.98) → 43.71 (49.43)	20.86 (18.40) → 40.91 (62.83)	.30
ALP	286.43(226.26) → 129.40 (16.67)	319.30 (237.57) → 131.38 (14.90)	.19

‡ Chi- square test

† Paired t-test

ESRD, end stage renal disease. ADPKD, autosomal dominant polycystic kidney disease. ATN, acute tubular necrosis. DGF, delayed graft function. Lab data, laboratory data. Hb, hemoglobin. WBC, white blood cell. PLT, platelet. AST, aspartate aminotransferase. ALT, alanine aminotransferase. ALP, alkaline phosphatase

**Fig. 1:**
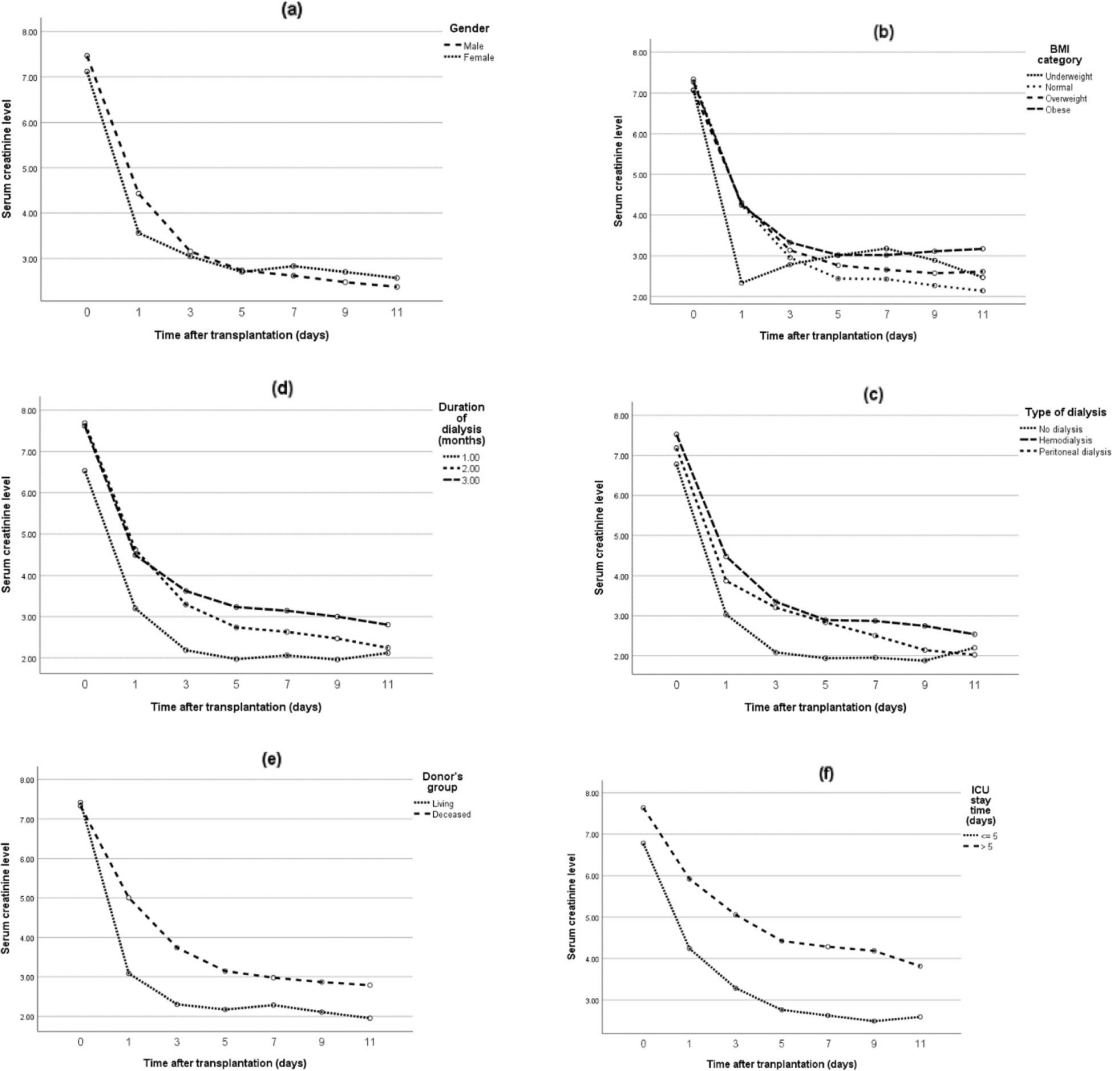
The relation between early postoperative creatinine trend and non-surgical variables*: a. gender (*p* = 0.734), b. body mass index (*p* = 0.768), c. type of dialysis (*p* = 0.036), d. duration of dialysis (*p* = 0.004), e. type of donor (*p* < 0.001), and f. length of ICU stay (p = 0.004) * Serum creatinine was measured every other day after transplant until discharge

**Fig. 2:**
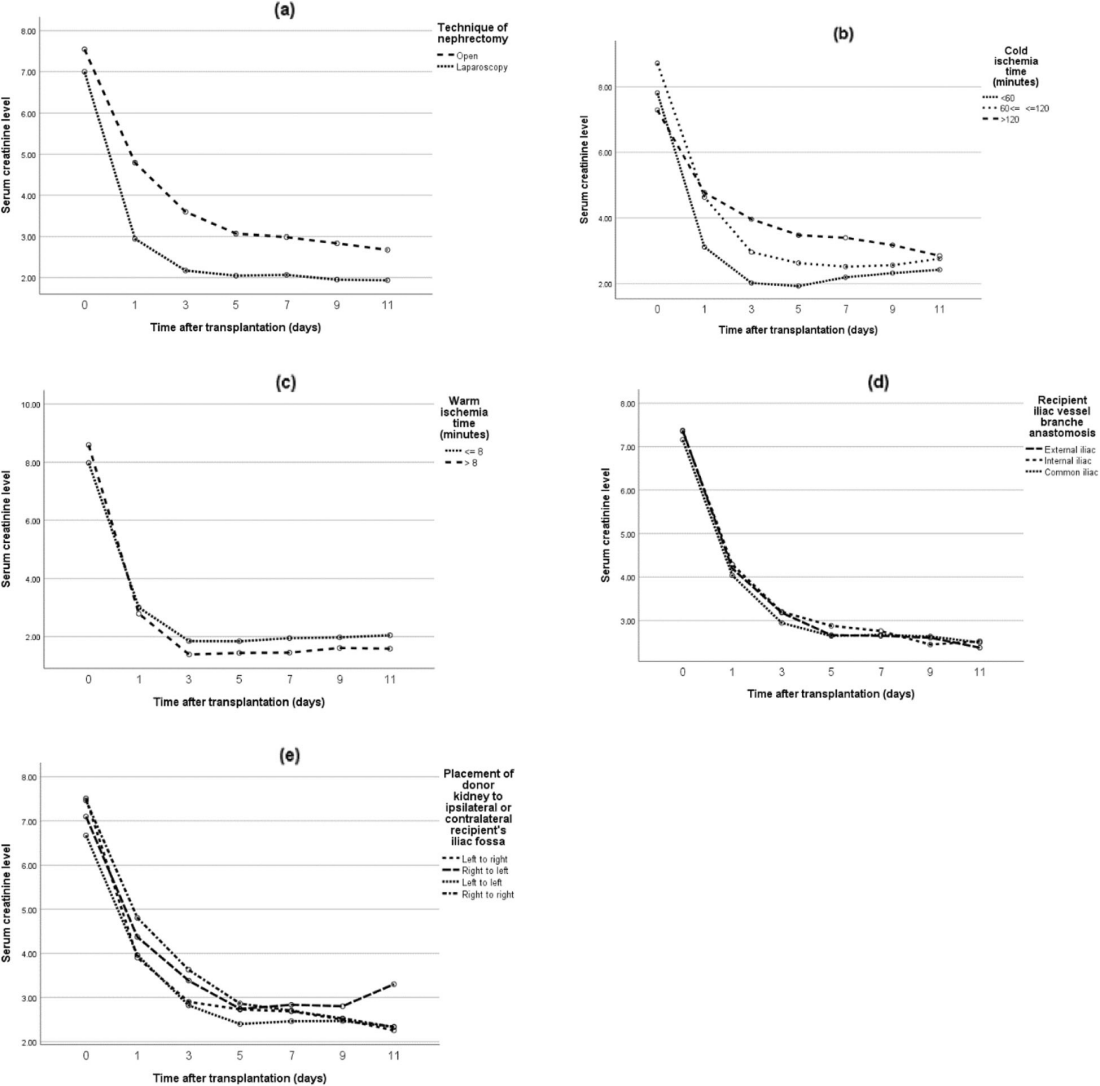
The relation between early postoperative creatinine trend and surgical variables*: a. techniques of donor nephrectomy (*p* = 0.001), b. cold ischemia time (*p* = 0.021), c. warm ischemia time (*p* = 0.768), d. method of iliac vessel anastomosis (*p* = 0.940), and e. ipsilateral or contralateral placement of the donor kidney (*p* = 0.740) * Serum creatinine was measured every other day after transplant until discharge

In addition, the effect of non-surgical and surgical variables on the early postoperative creatinine trend was investigated (Figs. 1 & 2). The multiple GEE model showed that donor type, duration of dialysis, and ICU stay of fewer than 5 days were predictive factors of post-transplant creatinine trend (*p* = 0.014, 0.012 and < 0.001, respectively) (Table 4). Also, by performing multiple linear regression analysis with backward elimination, the effect of these variables on the difference between serum creatinine before transplant and on the day of discharge was explored. In this regard, living-donor kidney transplant was associated with a significantly higher decrease in post-transplant creatinine levels as compared to deceased donors (*p* = 0.027). Moreover, patients who were on peritoneal dialysis experienced less improvement in post-transplant serum creatinine level compared to those without a history of dialysis, although this difference was not statistically significant (*p* = 0.076).

**Table 4:** Multiple analysis on the difference between serum creatinine before transplant and on the day of discharge

GEE model ^**a**^	β	CI for β		*p*-value
Type of donor				
Living donor	.800	.160	1.440	.014
Deceased donor (reference)				
Type of dialysis				
Peritoneal dialysis	.241	−.502	.984	.525
Hemodialysis	.399	−.145	.942	.151
No dialysis (reference)				
Cold ischemia time	.001	−.007	.009	.843
Dialysis duration	.016	.004	.028	.012
ICU stay duration	−.583	−.643	−.522	< 0.001
**Linear regression analysis**^**b, c**^	β	CI for β		p-value
Living donor (compared to deceased donor)	1.062	0.144	1.998	.027
Peritoneal dialysis (compared to no dialysis)	−1.233	−2.593	0.119	.076

Beta: regression coefficient, SE: standard of error

^a^ The dependent variable is creatinine across different time points

^b^ Variable(s) entered in the analysis included: donor type, type of dialysis, duration of dialysis, cold ischemia time and duration of ICU stay

^c^ The dependent variable is difference of serum creatinine before transplant and on the day of discharge

## Discussion

In this study, we investigated the role of patients’ socioeconomic and psychologic condition on electing deceased or living donors. Our results revealed that patients with higher education and income tended to receive transplants from living donors more frequently. This was similar to the majority of studies showing that access to the kidney transplant waiting list is affected by higher level of education and socioeconomic deprivation [2–4, 30–32]. In addition, the results of our study showed that patients with insomnia and anxiety mostly desired transplant from living donors while patients with depression symptoms had a tendency for deceased donors.

Generally, kidney transplant candidates are prone to significant psychological distress in the transplant wait-list period. Previous studies have shown that dialysis patients anticipating deceased-donor transplant suffer from anxiety and depression during the transplant wait-list period [33]. Depression is the most common psychiatric issue among dialysis patients, mainly due to presence of multiple stressors in their lives. In one study, patients on dialysis had stated that while waiting for a transplant, “they felt their life was on hold and they had a sense of isolation”, indicating the disparity and depression that patients on dialysis face [33]. Moreover, waiting for a deceased-donor transplant is associated with a great deal of uncertainty since both the timing and the outcome of the transplant are unknown. The unpredictable results of this operation can lead to an increased feeling of stress and anxiety. Regarding living**-**donor transplant, since the donor who is either a relative or someone with an altruistic motive for donating is usually known to the recipient, somewhat different experiences have been reported [33]. Thus, the dynamic of the relationship between the donor and the recipient alters the experience of waiting period. Also, in contrast to deceased-donor transplant, anticipating transplant from a living-donor is an active process with a definite outcome [33]. The findings of our study might be justified by the fact that anxious patients desire an immediate transplant with assured favorable outcome more than others. Although no study has compared the psychological status of deceased and living-donor recipients before transplant, a study in Iran showed that depression and anxiety are significantly higher in the deceased-donor group after transplant [34]. There have also been multiple studies comparing psychological status of Iranian patients maintained on dialysis with those who have already received transplants, generally showing a lower anxiety score among transplant recipients compared to hemodialysis patients [18, 19].

In general, early post-transplant surgical complications are classified into three groups: vascular, urologic, and others. Vascular complications remain the major concern after kidney transplant with a reported incidence of 2 to 15%. In a study by Ayvazoglu Soy et al., the incidence of vascular complications on 2594 kidney transplants (76% living donors and 24% deceased donors) was 2.1% [35], with the most common complication being renal artery stenosis (0.6%). In another study by Ammi et al. on 312 kidney transplants, a vascular complication rate of 16.0% was reported [36]. Regarding urologic complications, in a study by Rasiliti and colleagues, the prevalence was reported to be 13.1% in 297 deceased kidney transplants [37]. Duty et al. reported this rate to be approximately 9% [38]. In the present study, we found the overall rate of surgical complications to be 9.13%. Postoperative vascular and urologic complications were observed in 5.8 and 1.5% of patients, respectively. Perhaps the lower rate of urologic complications in this study was due to the reason that kidney transplant is solely performed by urologists in Iran while elsewhere it is mostly performed by general surgeons. Nevertheless, although post-operative surgical outcomes were not different across the two groups of recipients in this study, medical problems were significantly more common in recipients of deceased donors and a higher prevalence of delayed graft function, acute tubular necrosis, and myocardial infarction were seen in these patients. In a study by Biag et al. performed on 63 patients with a mean follow-up duration of 14.05 months, infectious complications were the most common post-transplant adverse event [39]. There are limited studies about comparison of early postoperative complications; however, unscheduled surgical operation, more comorbidities, and possibly, less medical care received by recipients of deceased donors might explain the higher rate of medical complications in this group.

According to previous studies, risk factors of failure to reduce early post-transplant serum creatinine level include gender, BMI, type of dialysis, duration of dialysis, length of ICU stays, cold and warm ischemia time, and type of donor [40–42]. Our results showed that living-donor transplant, ICU stay less than five days, cold ischemia time less than sixty minutes and hemodialysis or no history of dialysis before kidney transplant had a significant effect on post-transplant creatinine level. Also, among recipients of living donors, those who underwent laparoscopic nephrectomy technique had a better post-transplant creatinine trend.

Ours study also estimated the waiting time for kidney transplant in recipients of living donors (7.05 months) to be significantly less than that of deceased donors (11.27 months). Also, it seemed that living donor transplant in Iran renders less waiting time compared to other countries, possibly due to the active living donor program and high rate of deceased donation. The transplant waiting time in the USA is almost 4.72 years for recipients of deceased donors and in European countries, this time is about 2 to 5 years [43]. However, in Norway, where living donation is more frequent, the waiting time is 11 months [44].

Although this was not the intention of our study, we found that hypertension and diabetes mellitus were the most common factors attributing to end-stage renal disease (ESRD) in our study population. In line with this finding, the United States Renal Data System (USRDS) has reported diabetes mellitus and hypertension as the major causes of (overall 63%), followed by glomerular diseases (14%) [45]. However, there was a considerable difference in the prevalence of urologic etiologies (13.45%) in our study compared to the data of USRDS. This may be due to the reason that centers included in this study were referral transplant centers admitting recipients who are more complicated cases.

There were some limitations to our study:

1. Our socioeconomic data was based on self-reported questionnaires which may have been inaccurately reported.
2. The reported psychological status of patients might be affected by the method of assessment, for example, results might have been different based on the person who interviewed the patients (psychiatrist, psychologist, or unspecialized medical staff). In this study, the psychological questionnaires were filled in by an independent blind interviewer who were medical staff.
3. Some outcomes required a greater number of patients to reach a more precise conclusion. For example, vascular complications are not common, thus for a better comparison of this outcome in the two groups of recipients, a larger sample size would have been beneficial [46].
4. Data about human leukocyte antigen (HLA) matching was missing in our study. Since HLA- mismatch affects the outcome of transplant and the post-transplant creatinine trend, we could not reach a definite conclusion in this regard [40].

## Conclusion

This study reveals novel information about the existing psycho-socioeconomic disparities in Iran in terms of access to type of donor and the medical and surgical outcomes associated with each type of transplant. In summary, higher educational level, higher monthly income, and suffering from anxiety and sleep disorders (based on the GHQ-28) influenced the choice of selecting a living-donor transplant, which came with the benefit of less waiting time and better early post-transplant creatinine trend. Moreover, although post-operative surgical outcomes were not different across the two groups of recipients, medical complications occurred considerably less in the living-donor group. However, since the results associated with outcome of transplant might change over time in both groups, follow-up of these patients would be beneficial. Also, future studies to compare the psychological status of these two group of recipients after transplantation is recommended.

The findings of this comprehensive multicenter study will provide evidence for policymakers to implement strategies and specific actions for providing support to susceptible individuals who are either socioeconomically or psychologically less advantaged. Hopefully, this will allow for an equal opportunity to choose between living or deceased-donor transplant.

## Supplementary information

**Additional file 1: Supplementary Table 1.** Univariable logistic regression to determine predictive factors for selecting type of donor.

## Data Availability

The datasets during and/or analyzed during the current study available from the corresponding author on reasonable request.
