# Supplementary material for: Living or deceased-donor kidney transplant: the role of psycho-socioeconomic factors and outcomes associated with each type of transplant
Source: Int J Equity Health. 2020 Jun 1;19:79. doi: 10.1186/s12939-020-01200-9 (PMC7268666; doi:10.1186/s12939-020-01200-9)
Supplement: Supplementary file 1 — Additional file 1: Supplementary Table 1. Univariable logistic regression to determine predictive factors for selecting type of donor. [file 12939_2020_1200_MOESM1_ESM.docx]

|  | **Crude OR** | **p-value** | **95% CI for OR** | |
| --- | --- | --- | --- | --- |
|  |  |  | **Lower** | **Upper** |
| **Age** | 1.008 | 0.178 | .996 | 1.021 |
| **Gender (reference: male)** | 0.806 | 0.306 | .533 | 1.218 |
| **Educational (reference: illiterate)** |  |  |  |  |
| Elementary to high school | 1.353 | 0.333 | .733 | 2.497 |
| Diploma | 1.141 | 0.660 | .634 | 2.055 |
| Academic | 3.279 | 0.001 | 1.604 | 6.702 |
| **Monthly income (reference: low income group)** |  |  |  |  |
| Lower-middle income | 2.872 | <0.001 | 1.806 | 4.567 |
| Upper-middle income | 3.423 | 0.002 | 1.596 | 7.340 |
| **Ethnicity (reference: other)** | 0.718 | 0.114 | .476 | 1.082 |
| **Financial support (reference: yes)** | 1.162 | 0.486 | .762 | 1.771 |
| **GHQ2 (reference: without anxiety and sleep disorder)** | 1.647 | 0.045 | 1.011 | 2.682 |
| **GHQ4 (reference: without depression symptoms)** | 0.619 | 0.045 | .386 | .990 |
| **SF-36: Physical component summary** | 1.005 | 0.281 | .996 | 1.013 |
| **SF-36: Mental component summary** | 1.001 | 0.737 | .997 | 1.002 |
| **SSQ family (reference: low+ med)** | 0.947 | 0.858 | .522 | 1.718 |
| **SSQ friend (reference: low+ med)** | 0.882 | 0.557 | .579 | 1.342 |
| **SSQ entourage (reference: low+ med)** | .825 | 0.552 | .438 | 1.555 |
| **ESRD causes (reference: DM+HTN)** | 1.148 | 0.493 | .774 | 1.703 |

**Supplementary Table 1.** Univariable logistic regression to determine predictive factors for selecting type of donor.

OR, odds ratio. CI, confidence interval. DM, diabetes mellitus. HTN, hypertension. GHQ, General Health questionnaire. SF-36, Short Form Health Survey-36 SSQ, Social support questionnaire. ESRD, End stage renal disease.
